# Cadmium, an Environmental Contaminant, Exacerbates Alzheimer’s Pathology in the Aged Mice’s Brain

**DOI:** 10.3389/fnagi.2021.650930

**Published:** 2021-06-24

**Authors:** Tahir Ali, Amjad Khan, Sayed Ibrar Alam, Sareer Ahmad, Muhammad Ikram, Jun Sung Park, Hyeon Jin Lee, Myeong Ok Kim

**Affiliations:** ^1^Division of Applied Life Science (BK 21 Four), College of Natural Science, Gyeongsang National University, Jinju, South Korea; ^2^Calgary Prion Research Unit, Department of Comparative Biology & Experimental Medicine, Faculty of Veterinary Medicine, Hotchkiss Brain Institute Cumming School of Medicine, University of Calgary, Calgary, AB, Canada

**Keywords:** Cadmium, reactive oxygen species, antioxidant genes Nrf-2/HO-1, Alzheimer’s disease, neurodegeneration

## Abstract

Cadmium (Cd) is an environmental contaminant, which is a potential risk factor in the progression of aging-associated neurodegenerative diseases. Herein, we have assessed the effects of chronic administration of Cd on cellular oxidative stress and its associated Alzheimer’s disease (AD) pathologies in animal models. Two groups of mice were used, one group administered with saline and the other with Cd (1 mg/kg/day; intraperitoneally) for 3 months. After behavioral studies, molecular/biochemical (Immunoblotting, ELISAs, ROS, LPO, and GSH assays) and morphological analyses were performed. We observed an exacerbation of memory and synaptic deficits in chronic Cd-injected mice. Subacute and chronic Cd escalated reactive oxygen species (ROS), suppressed the master antioxidant enzymes, e.g., nuclear factor-erythroid 2-related factor 2 and heme oxygenase-1, and evoked the stress kinase phospho-c-Jun N-terminal kinase 1 signaling pathways, which may escalate AD pathologies possibly associated with amyloidogenic processes. These findings suggest the regulation of oxidative stress/ROS and its associated amyloid beta pathologies for targeting the Cd-exacerbated AD pathogenesis. In addition, these preclinical animal studies represent a paradigm for epidemiological studies of the human population exposed to chronic and subacute administration of Cd, suggesting avoiding environmental contaminants.

## Introduction

Alzheimer’s disease (AD) is a progressive age-associated brain disorder. AD significantly becomes prevalent with the aging of the world’s population, and the world is opposing a great challenge, the era of aging. Thus, aging is one of the biggest risk factors of neurodegenerative diseases such as AD and its associated dementia (Livingston et al., 2019). The synergistic effects of environmental factors, genetic factors, and lifestyle accelerate memory defects and contribute to the progression and onset of neurodegenerative diseases (Engstrom et al., 2017; Moser and Pike, 2017; Newcombe et al., 2018). Globally, overwhelming evidence indicated that environmental factors are the main factors that induced the pathology of neurodegenerative diseases. In environmental factors, metals are the key neurotoxic chemicals that induce impaired homeostasis in the olfactory neurons and the brain. The detrimental effects of metals on the brain are closely linked with progression of neurodegenerative disorders including AD (Mascagni et al., 2003; Kim et al., 2018; Newcombe et al., 2018; Huat et al., 2019).

Cadmium (Cd) is one of the major environmental contaminants, which exists in various foods, mostly in cereals, vegetables, root crops, seafood, and debris of meat, and most importantly in tobacco smoke. Of note, consumption of tobacco products or inhalation of tobacco smoke increases the risk of Cd-related morbidities or vice versa and other morbidities in the general population (Sanders et al., 2015; Kippler et al., 2016; Richter et al., 2017; Ganguly et al., 2018; Gustin et al., 2018). From the last few years, several epidemiological studies reported that people at early-age exposure to Cd have been observed for alterations in cognition deficits (Sanders et al., 2015; Gustin et al., 2018). Additionally, a huge number of epidemiological studies reported that environmental exposure to Cd may implicate in the etiology of other fatal chronic diseases, e.g., kidney, cardiovascular diseases, cancer, and liver diseases (Horiguchi et al., 2013; Li et al., 2016; Zhou et al., 2016; Satarug et al., 2020). Recently, the accumulation of Cd in the brain has been documented, where it acts as a potent neurotoxin (Min and Min, 2016; Peng et al., 2017). However, these epidemiological studies would require proof of concept and evidence research-based investigation in age-associated preclinical animal models. From the last few years, several *in vitro* and *in vivo* studies have been designed to investigate the detrimental effects of chronic Cd in the brain. Nevertheless, the comprehensive underlying cellular impairments yet remain elusive in the Cd-induced memory deficits of phenotype (Shukla et al., 1996; Antunes and Biala, 2012; Akinyemi et al., 2017; Favorito et al., 2017; Larner et al., 2017; Wang et al., 2018; Pulido et al., 2019). Previous studies have suggested that Cd can cross the blood-brain barrier (BBB) and may accumulate in the brain tissue and elicit severe neurotoxicity (Wang and Du, 2013). In the brain, Cd induces activation of inflammation, oxidative stress, and neuronal apoptosis (Chen et al., 2008; Xu et al., 2011; Yuan et al., 2018). Recent epidemiological studies reported that blood Cd levels were significantly associated with AD-related mortality among older adults (Min and Min, 2016; Peng et al., 2017). However, these cellular and molecular mechanisms remain elusive; very few studies have been conducted on the role of Cd in inducing age-related neurodegenerative conditions and AD-like pathological changes in the brain.

Numerous studies reported that Cd acts as a potent reactive oxygen species (ROS) and oxidative stress inducer which mediated other signaling and cellular homeostasis and consequently led to neurodegeneration. Antioxidants nuclear factor-erythroid 2-related factor 2 (Nrf2) and heme oxygenase-1 (Nrf2/HO-1) are key regulators of the antioxidant response and play a role in protecting cells against oxidative stress (Ma, 2013; Loboda et al., 2016; Cores et al., 2020). Under normal conditions, Nrf2 is present in the cytoplasm in an inactive state with its inhibitory protein, kelch-like ECH-associated protein 1 (Keap-1; Tkachev et al., 2011). Several factors may disrupt this association causing the dissociation of Nrf2 from kepa-1 and translocating Nrf2 to the nucleus (Kensler et al., 2007), causing the induction of other genes such as HO-1 (Numazawa et al., 2003). Similarly, activated ROS/oxidative stress triggers mitogen-activated protein kinases (MAPKs), including phospho-c-Jun N-terminal kinase 1 (p-JNK1). Further, the cross talk and correlation between Nrf2 and activated p-JNK have been widely described in neuroinflammation and neurodegeneration (Ali et al., 2018; Lee et al., 2019; Cores et al., 2020). The suppression of Nrf2 and activation of *p*-JNK have aggravated the inflammatory events and amyloidogenesis (Ahmed et al., 2017; Rehman et al., 2018; Bahn et al., 2019). Herein, our aim is to investigate the detrimental effects of subacute and chronic Cd administration on accumulation of oxidative stress/ROS, Nrf2/HO-1, and activated p-JNK which might be associated with increased amyloid β (Aβ) level and hence possibly develop a feedforward mechanism which subsequently exacerbates AD pathologies in the aged mice’s brain.

## Materials and Methods

### Materials and Chemicals

Cadmium chloride, 2′7′-dichlorodihydrofluorescein diacetate (DCFH-DA), 3,3-diaminobenzidine tetrahydrochloride (DAB), and dimethyl sulfoxide (DMSO) were purchased from Sigma Chemical Co. (St. Louis, MO, USA). Lipid peroxidation (LPO assay) kit catalog # K739-100) and glutathione (GSH) assay kit catalog # K264-100 were obtained from BioVision corporation (A 95035 USA). The Aβ42 ELISA kit was obtained from Invitrogen, Thermo Fisher Scientific (Rockford, IL, USA) Dulbecco’s modified Eagle’s medium (DMEM) was obtained from Gibco Life Sciences. The chamber slides were obtained from Thermo Fisher Scientific (75 Panorama Creek Drive Rochester, NY14625-2385, USA).

### Mice, Housing Environment, and Ethics Statement

The mice used for experimental purposes were old male wild-type C57BL/6N mice (44–50 g, 16 months old) and were kept in the university animal housing at the 12-h/12-h light/dark cycle and a maintained temperature (23°C) with humidity environment 60 ± 10% in a separate and pathogen-free room. The mice were allowed free access to normal food and water *ad libitum*. For the animal care and treatments in this animal model, we have followed the guidelines (Approval ID: 125) issued by the ethics committee (IACUC), Division of Applied Life Sciences, Gyeongsang National University, South Korea. All the experiments were performed according to the established ARRIVE protocols and guidelines.

### Establishing Aging and Environment Factor (Cd) Interaction Preclinical Animal Model

**Figure 1:**
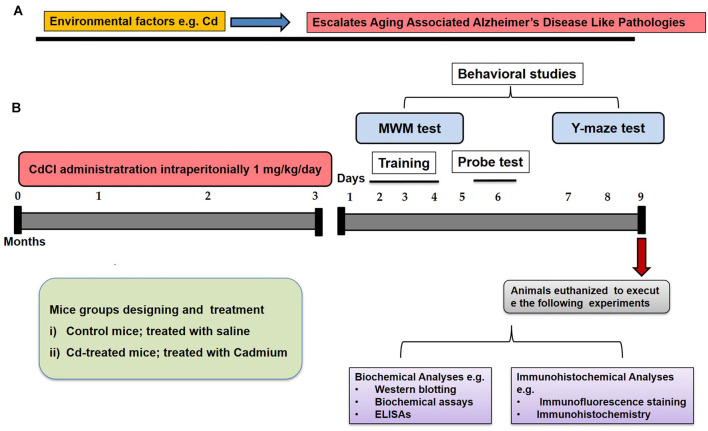
Schematic diagram. **(A)** These schematic illustrations show our hypothesis for interaction and synergistic effects of an environmental factor (Cd) and aging. **(B)** The illustrative graphical representation indicated our study designing to establish the control of aging and Cd-induced aging models. The treatment paradigms, behavioral studies [Morris water maze (MWM) and Y-maze tests], animal authorization, and biochemical as well as immunohistochemical representation were indicated for the entire study.

We selected environmental factor (Cd) as an interactive model with an aging preclinical model to escalate brain degeneration (Figure 1A). To execute our hypothesis and experiments, we aimed to include two groups in our study (Figure 1B). The old mice of 16 months (12/group) were randomly divided into the following two groups and throughout the treatment paradigm, and the animals were handled very carefully to minimize the suffering and stress conditions.

(i) The mice were treated with saline as a vehicle for 3 months (Cont-).
(ii) The mice were treated with Cd as an environmental factor at a dose of 1 mg/kg for 3 months (Cd-).

Cd in its salt form cadmium chloride was dissolved in normal saline and administered at the dose of 1 mg/kg/day, intraperitoneally (I.P) for 3 months. Similarly to the control group, the same amount of saline was administered for 3 months under the same conditions.

### The Evaluation of Learning and Memory Functions

To assess the learning and memory functions in the Cont- and Cd- mice, we performed behavioral assays [Morris water maze (MWM) and a Y-maze test] after the completion of chronic treatment of saline and Cd. The mice were carried out to a separate and control behavioral room, and experiments were conducted with careful protocols according to universal ethical guidelines.

#### MWM Test

The MWM test is a parameter task to evaluate memory functions. The experimental apparatus consisted of a circular water tank (100 cm in diameter, 40 cm in height) containing water (23 ± 1°C) to a depth of 15.5 cm, which was rendered opaque by adding white paint. A transparent escape platform (10 cm in diameter, 20 cm in height) was hidden 1 cm below the water surface and placed at the midpoint of one quadrant. After 3 months of treatment completion, the MWM test was started as 1st day and completed on the 6th day (Figure 1B). Each mouse received training each day for four consecutive days using a single hidden platform in one quadrant with three rotating starting quadrants. Latency to escape from the water maze (finding the submerged escape platform) was calculated for each trial. The probe test was performed by removing the platform and allowing each mouse to swim freely for 60 s. The time the mice spent in the target quadrant (where the platform was located during hidden platform training) was measured. The time spent in the target quadrant is considered to represent the degree of memory consolidation that has taken place after learning. All data were recorded using video-tracking software (SMART, Panlab Harvard Apparatus Bioscience Company, USA).

#### Y-Maze Test

The Y-maze was constructed from painted black wood. Each arm of the maze was 50 cm long, 20 cm high, and 10 cm wide at the bottom and top. After the MWM test, the Y-maze was initiated on day 7 and completed on day 9 (Figure 1B). On day 7, the mice were habituated with Y-maze environment. On day 8, we used the training session three times a day, and then on day 9 we did the final test using a three-time session for all mice. Each mouse was placed at the center of the apparatus and allowed to move freely through the maze for three 8-min sessions. The series of arm entries was visually observed. The spontaneous alternation was defined as the successive entry of the mice into the three arms in overlapping triplet sets. Alteration behavior (%) was calculated as follows: [successive triplet sets (entries into three different arms consecutively)/total number of arm entries-2] × 100.

### Mouse Euthanization and Protein Extraction From the Mouse Brains for Immunoblotting and Various Biochemical Assays

Following the completion of behavioral assays, the mice were brought to a separate control surgical room. The mice were euthanized with cervical dislocation (approved from the institute ethical committee) without anesthesia because anesthesia decreased the body temperature of the animals, which might interfere with finding and inducing artificial tau phosphorylation and other cellular signaling impairment (Ali et al., 2015). Instantly, the brains were collected and incubated in 1% RNA later, and cortical and hippocampal tissues were carefully separated. The collected cortical and hippocampal tissues were stored at −80°C until further processing. The cortical and hippocampal tissues were homogenized in 0.2 M phosphate-buffered saline (PBS) with a phosphatase inhibitor and protease inhibitor cocktail and then centrifuged at 10,000 *g* at 4°C for 25 min. The supernatants were collected and stored at −80°C until processing. After preliminary confirmation in individual cortical and hippocampal mouse brain protein, the protein was further pooled from seven mice for each tissue per group (none of the mice were excluded) for immunoblotting and other biochemical assays and ELISAs.

### The ROS Assay in the Cortical and Hippocampal Brain Homogenates

The ROS assay was based on the oxidation of DCFH-DA to 2′,7′-dichlorofluorescein (DCF). Subsequently, cortical and hippocampal homogenates were diluted with ice-cold Lock’s buffer at a ratio of 1:20 to maintain 2.5 mg tissue/500 ml final concentration. The reaction mixture of Lock’s buffer (1 ml, pH = 7.4), 0.2 ml homogenate, and 10 ml of DCFH-DA (5 mM) was incubated at room temperature for 15 min to convert DCFH-DA to the fluorescent product DCF. The conversion of DCFH-DA to DCF was performed using a spectrofluorometer with excitation at 484 nm and emission at 530 nm. For background fluorescence assessment (conversion of DCFH-DA in the absence of homogenate), we ran parallel blanks. The quantification analysis of ROS is expressed as pmol DCF formed/mg protein in cortical and hippocampal homogenates.

### The LPO Analysis in the Cortical and Hippocampal Brain Homogenates

According to the manufacturer’s protocols and guidelines, the LPO amount was examined in the cortical and hippocampal (*n* = 7 mice per group) homogenates through assessing the level of malondialdehyde (MDA), a biomarker of LPO.

### The GSH Analysis in the Cortical and Hippocampal Brain Homogenates

According to the manufacturer’s protocols and guidelines, the GSH amount was examined in the cortical and hippocampal (*n* = 7 mice per group) homogenates using the commercially available GSH assay kit.

### The *In vitro* Culture and Drug Treatment

The immortalized murine hippocampal (HT22) neuronal cells were cultured in 35-cm^2^ plates (Thermo Scientific, Nunc™ EasYFlask™ 75-cm^2^ Nunclon™ Delta surface, Thermo Fisher Scientific A/S, Kamstrupvej 90. P.O. Box 280 DK-4000 Roskilde, Denmark), and the numbers of astrocytes and HT22 cells were counted using a disposable hemocytometer (the full grid on a hemocytometer contained nine squares of 1 mm^2^ each) through the addition of 10 ml of the media that contained the cells to both sides of the hemocytometer chamber. Four 1/25-mm^2^ corners and the middle central square were used to count the cells under 10× magnification using an Olympus IX70 microscope. The average number of cells on both sides of the chamber was calculated. The cells (2 × 10^4^/ml) were further subcultured in chamber slides (Thermo Fisher Scientific 75 Panorama Creek Drive Rochester, NY14625-2385, USA) in DMEM supplemented with 10% FBS and 1% antibiotics at 37°C in humidified air containing 5% CO_2_. After the cells reached 50–80% confluence, they were treated with Cd (10 μM) and a JNK-specific inhibitor SP600125 (20 μM). The cells in the control group were treated with DMSO (0.01%) because we dissolved the SP600125. After a 12-h drug treatment, cells were washed with 0.01 M PBS and fixed with 4% paraformaldehyde and kept at −80°C until processing for immunofluorescence and immunohistochemical analyses.

### Western Blot Analysis

Immunoblotting was performed as previously described (Badshah et al., 2015; Ali et al., 2018). Briefly, the protein concentrations for both cortical and hippocampi were measured through a Bio-Rad protein assay kit (Bio-Rad Laboratories, CA, USA). Equal amounts of protein (10–30 μg) underwent sodium dodecyl sulfate-polyacrylamide gel electrophoresis (SDS-PAGE) using 4–12% Bolt™ Mini Gels (Novex, Life Technologies). The membranes were blocked in 5% (w/v) skim milk to reduce nonspecific binding and incubated with primary antibodies (details available in Table 1) overnight at 4°C. After undergoing a reaction with a horseradish peroxidase-conjugated secondary antibody (goat anti-mouse IgG, goat anti-rabbit IgG, and rabbit anti-goat IgG were purchased from Santa Cruz Biotechnology), as appropriate, the proteins were detected using an ECL detection reagent according to the manufacturer’s instructions (Amersham Pharmacia Biotech, Uppsala, Sweden). Then, X-ray films were scanned, and the optical densities of the bands were analyzed through densitometry using the computer-based Sigma Gel program, version 1.0 (SPSS, Chicago, IL, USA).

**Table 1:** The list of primary antibodies and their detailed information.

Antibody	Host	Application	Manufacturer	Catalog Number	Concentration
Nrf-2	Rabbit	WB/IF	Santa Cruz Biotechnology	SC 722	1:1,000/1:100
HO-1	Mouse	WB/IF	Santa Cruz Biotechnology	SC 136961	1:1,000/1:100
Aβ	Mouse	WB/IF	Santa Cruz Biotechnology	SC-28365	1:1,000/1:100
Iba-1	Rabbit	WB	Santa Cruz Biotechnology	SC 98468	1:1,000
GFAP	Mouse	WB/IF	Santa Cruz Biotechnology	SC 33673	1:1,000/1:100
PSD-95	Mouse	WB	Santa Cruz Biotechnology	SC 71933	1:1,000
Synaptophysin	Rabbit	IF	Santa Cruz Biotechnology	SC 17750	1:100
Aβ (6E10)	Mouse	IF	Covance, CA, USA	SIG-39300	1:100

*IF, immunofluorescence; WB, Western blotting*.

### The Mouse Perfusion and Brain Tissue Preparation for Morphological Assessment

We performed the perfusion in a separate control room in which the heating system was designed to control the body temperature maintained at 36°C–37°C (Badshah et al., 2014; Ali et al., 2015). The mice were first perfused transcardially with 0.9 saline solution and then with 4% ice-cold paraformaldehyde, and brains were postfixed for 72 h in 4% paraformaldehyde and transferred to 20% sucrose for 72 h. Then, brains were frozen in O.C.T. compound (A.O, USA), and 14-μm coronal sections were cut using a CM 3050C cryostat (Leica, Germany). The sections were thaw-mounted on ProbeOn Plus charged slides (Fisher, USA).

### Immunofluorescence Staining and Confocal Microscopy Analysis

Immunofluorescence was performed as previously described (Ahmad et al., 2016; Khan et al., 2017). Briefly, the slides carrying brain tissue and chamber slides containing cells were washed twice for 10 min in 0.01 M PBS, followed by incubation for 1 h in blocking solution containing 2% normal serum according to the antibody treatment and 0.3% Triton X-100 in PBS. After blocking, the slides were incubated overnight at 4°C in the primary antibodies, diluted 1:100 in blocking solution. Following incubation with primary antibodies (mentioned in Table 1), the sections were incubated for 2 h in fluorescein isothiocyanate (FITC) secondary antibodies and tetramethylrhodamine isothiocyanate (TRITC)-labeled antibodies (1:500; Santa Cruz Biotechnology). The slides were mounted with ProLong Antifade Reagent (Molecular Probe, Eugene, OR, USA). Staining patterns of the double immunofluorescence and single immunofluorescence were examined using a confocal laser-scanning microscope (CLSM; FlouView FV 1000). Five images per section (tissue) were captured (by the operator blinded to the experimental groups) from every respective group. Next, the real confocal images were changed to the tagged image file format (TIF). Quantification of immunofluorescence intensity in the same region of the cortex/total area and hippocampus/total area of TIF images for all groups was measured using ImageJ software using the method described as follows. The TIF image background was optimized according to the threshold intensity, and the immunofluorescence intensity was analyzed at the specified threshold intensity for all groups and was expressed as the integrated density of the samples relative to the control.

### The ELISAs in the Cortical and Hippocampal Brain Homogenates

According to the manufacturer’s protocols and guidelines, the cortical and hippocampal homogenates as well as cell lysates where appropriate were processed for the Aβ_1–42_ through Elisa analysis.

### Statistics

The obtained immunoblot results/images were scanned and quantified *via* densitometry analyses using the Sigma Gel System (SPSS Inc., Chicago, IL, USA). Density values were expressed as the mean ± standard error of the mean (SEM). For immunofluorescence and immunohistological image quantification, we used ImageJ software. The statistical analyses and histogram representation were done through GraphPad Prism 8 using one-way analysis of variance (ANOVA) followed by two-tailed independent Student’s *t*-test and Tukey test where applicable. The data are presented as the means ± SEM of the three independent biological and reproducible experiments. Statistical significance = *P* < 0.05.

## Results

### Cd-Exposure Accelerated Memory Impairment in Mice

**Figure 2:**
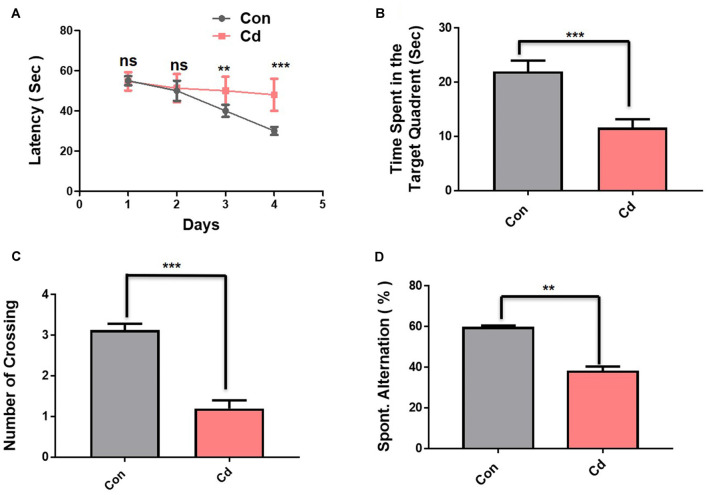
Cd exposure accelerated memory impairment in mice. The behavioral studies were performed through MWM and the Y-maze test. The mice (12 mice per group) were used for the behavioral analysis. **(A)** The time it took [escape latency (sec)] to reach the submerged hidden platform during training. **(B)** The graphs represent the duration spent in the target quadrant (where the platform was located during the hidden platform training session) during the probe test. **(C)** The number of platform crossings during the probe test. **(D)** The graphs represent the % of spontaneous alternation behavior in the Y-maze test. The escape latencies in the behavioral tests were analyzed using a two-way ANOVA, with training days as the repeated measurements, while for other probe tests and Y-maze test, the Student’s unpaired *t*-test was used. The graphs express the means ± standard error of the mean (SEM; *n* = 12 mice/group). Significance = **p < 0.01, ***p < 0.001; ns = non-significant.

The interaction between environment, genetic, and lifestyle factors induced accumulative effects and mediated AD pathology (Engstrom et al., 2017; Moser and Pike, 2017; Newcombe et al., 2018). Considering the environmental factor’s effect on aging, we chose the most potent environmental ubiquitous contaminant Cd and evaluate its chronic treatment (1 mg/kg, i.p., for 3 months) effect on the mouse brain. Subsequently, on termination of treatment, we performed MWM and Y-maze test (Figure 1B). In the MWM test, the observed latency time to reach the hidden platform during a training session for 4 days indicated that comparative to control mice the Cd-treated mice took a long time get to reach the hidden platform (Figure 2A). Next, after a 1-day interval, the hidden platform was taken and probe testing was carried out. The results of the probe task show that mice treated with Cd spent very little time in the target quadrant where the hidden platform was placed (Figure 2B). The mice treated with Cd have also been observed significantly with less number of platform crossing (Figure 2C). To assess spatial working memory functions, we performed the Y-maze test after the execution of the MWM test. The results of the Y-maze show that Cd-exposed mice showed less spontaneous alteration behavior in % compared to control mice, revealing that Cd impaired spatial memory functions (Figure 2D).

### Cd Exposure Induced Synaptic Dysfunction in Mice

**Figure 3:**
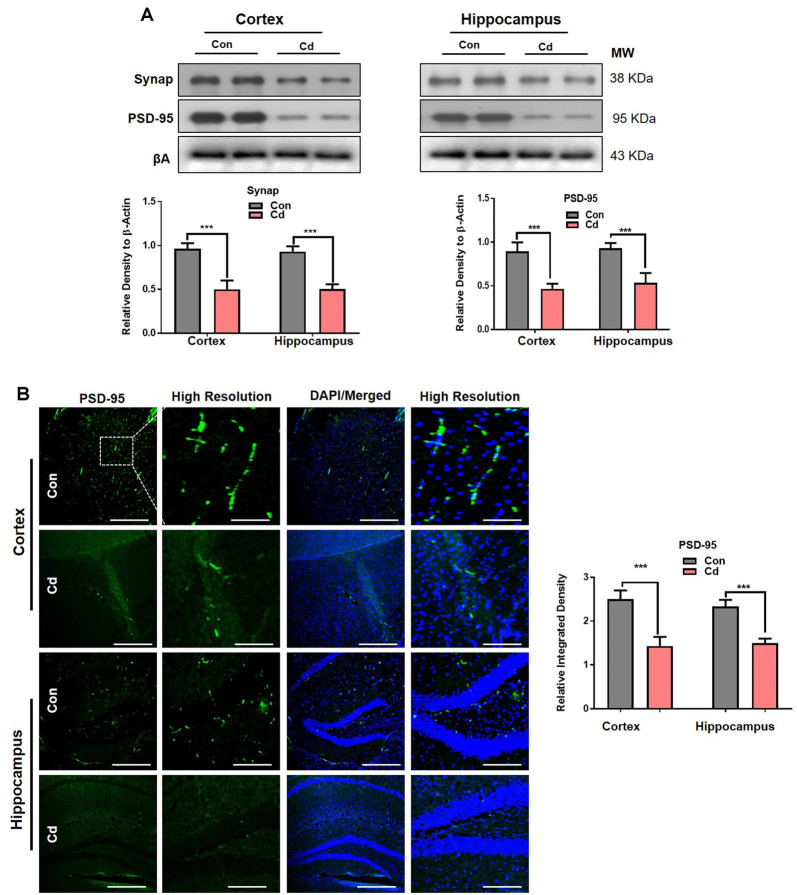
Cd exposure induced synaptic dysfunction in mice. **(A)** Western blot images against presynaptic (Synap) and postsynaptic (PSD-95) protein markers in the cortical and hippocampal regions of the brain and their respective histograms. β-Actin was used as a loading control. The histograms represented as the means ± SEM (*n* = 7 mice/group) for three independent repeated and reproducible experiments. Significance = ***p < 0.001; Student’s unpaired *t*-test. See Supplementary Figure 1 for uncropped and original blots. **(B)** Representative immunofluorescence images of PSD-95, respectively (green: FITC and blue: DAPI) in the cortical and hippocampal regions of the mouse brain. The histograms represented as the means ± SEM for *n* = 5 mice per group, and the number of independent experiments = 3. Magnification: 10×. Scale bar = 50 μm. Significance = ***p < 0.001; Student’s unpaired *t*-test.

To confirm the behavioral results, we examined biochemically and immunohistochemically the main pre- and postsynaptic protein markers implicated in memory functions. The immunoblotting indicated that Cd exposure significantly reduced the presynaptic (synaptophysin; Synap) and postsynaptic (postsynaptic density; PSD-95) markers in the brain of mice compared to the control mice (Figure 3A). The confocal results also confirmed that chronic Cd remarkably reduced immunofluorescence reactivity of Synap and PSD95 in the Cd-treated mice compared to control mice (Figure 3B). Together, these results supported that environmental factors (Cd) aggravate the aging risk factor and expedite the memory and synaptic impairment of aging mice.

### Cd Exposure Accumulated Oxidative Stress/ROS

**Figure 4:**
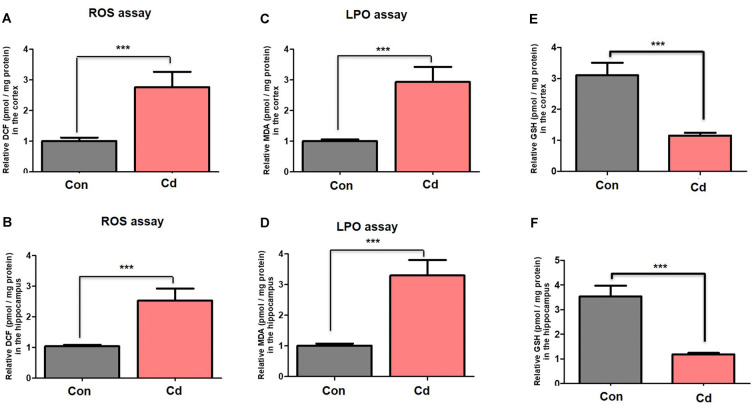
Chronic Cd exposure escalated oxidative stress/ROS accumulation. **(A,B)** Histograms indicated results of ROS assay in the cortical and hippocampal regions of the mouse brain. The results were represented as the means ± SEM (number = 7 mice/group) for three repeated independent and reproducible experiments. Significance = ***p < 0.001; Student’s unpaired *t*-test. **(C,D)** Histograms indicated results of LPO assay and MDA analyses in the cortical and hippocampal regions of the mouse brain. The results were represented as the means ± SEM (number = 7 mice/group) for three repeated independent and reproducible experiments. Significance = ***p < 0.001. Student’s unpaired *t*-test. **(E,F)** Histograms indicated results of GSH assay in the cortical and hippocampal regions of the mouse brain. The results were represented as the means ± SEM (number = 7 mice/group) for three repeated independent and reproducible experiments. Significance = ***p < 0.001; Student’s unpaired *t*-test.

Cellular homeostasis impairment related to excessive oxidative stress and ROS accumulation leads to alteration in memory impairment and neuronal degeneration (Beckhauser et al., 2016). Further, other factors such as aging increase vulnerability to accumulated oxidative stress and ROS (Topic et al., 2016). Here, we observed the aggravated ROS and MDA accumulation through ROS and LPO assays in the brain of mice compared to the control mice (Figures 4A–D). Also, we found a significant reduction in the level of antioxidant enzyme GSH in the brain’s homogenates of chronic Cd-exposure mice compared to control mice (Figures 4E,F).

### Cd Exposure Perturbed Antioxidant Nrf2/HO-1 Pathways

**Figure 5:**
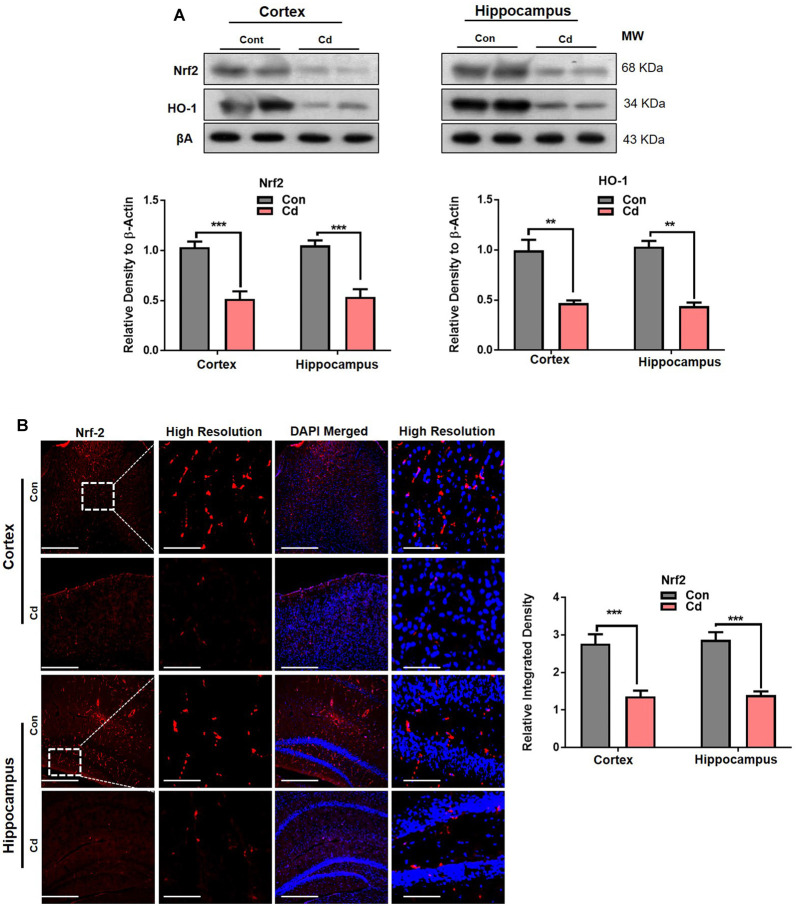
Cd exposure perturbed antioxidant Nrf2/HO-1 pathways. **(A)** The Western blot images against Nrf2 and HO-1 in the cortical and hippocampal regions of the brain and their respective histograms. β-Actin was used as a loading control. The histograms represented as the means ± SEM (*n* = 7 mice/group) for three repeated independent and reproducible experiments. Significance = **p < 0.01, ***p < 0.001; Student’s unpaired *t*-test. See Supplementary Figure 2 for uncropped and original blots.** (B)** Representative immunofluorescence images of Nrf2 (red: TRITC and blue: DAPI) in the cortical and hippocampal regions of the mouse brain. The histograms represented as the means ± SEM for *n* = 5 mice per group, and the number of independent experiments = 3. Magnification: 10×. Scale bar = 50 μm. Significance = ***p < 0.001.

The Cd exposure induced accumulated oxidative stress and ROS production, speculated to induce imbalance of the cellular homeostasis and perturb the antioxidant Nrf2/HO-1 pathways. Our results supported the previous studies and found that Cd treatment to the mice significantly abated the Nrf2 and HO-1 levels compared to the normal mouse brain (Figure 5A). The confocal microscopy results indicated reduced Nrf2 immunofluorescence reactivity in the Cd-treated mouse brain compared to the control mice (Figure 5B). These results unveiled that chronic Cd significantly aggravates the accumulation of oxidative stress/ROS and suppressed the endogenous antioxidant enzymes in the brain, suggesting that Cd exposure may further exacerbate the impairment in the cellular homeostasis of the brain.

### Cd Exposure Increased the p-JNK and BACE-1 Levels

**Figure 6:**
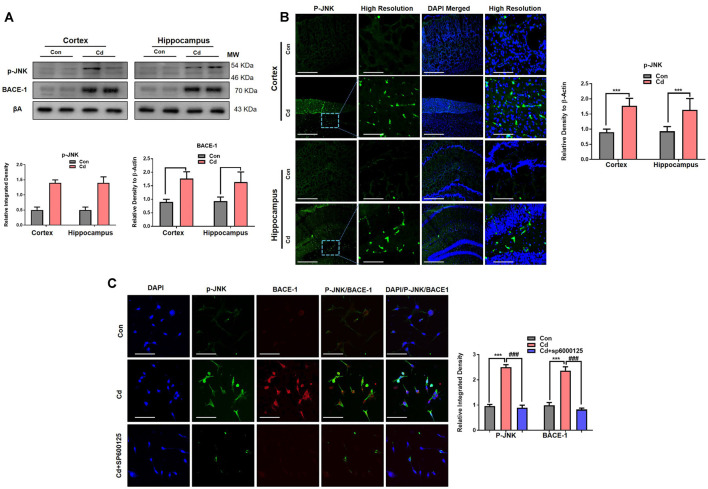
Cd exposure increased the expression of p-JNK and BACE-1. **(A)** Western blot images against p-JNK and BACE-1 in the cortical and hippocampal regions of the brain and their respective histograms. β-Actin was used as a loading control. The histograms represented as the means ± SEM (*n* = 7 mice/group) for three repeated independent and reproducible experiments. Significance = ***p < 0.001; ^###^*p* < 0.001 Student’s unpaired *t*-test. See Supplementary Figure 3 for uncropped and original blots. **(B)** Representative immunofluorescence images of p-JNK (green: FITC and blue: DAPI) in the cortical and hippocampal regions of the mouse brain. The histograms represented as the means ± SEM for *n* = 5 mice per group, and the number of independent experiments = 3. Magnification: 10×. Scale bar = 50 μm. Significance = ***p < 0.001; ^###^*p* < 0.001. **(C)** The double immunofluorescence and co-localization of p-JNK (green: FITC and blue: DAPI) and BACE-1 (red: TRITC and blue: DAPI) in the hippocampal neuronal HT22 cells exposed to Cd (10 μM) and SP600125 (20 μM). The data are indicated as the ±SEM for *n* = 5 images per group, and the number of independent experiments = 3.

The exposure of Cd impaired non-amyloidogenic (ADAM10 and neutral endopeptidase) processes of APP, which is responsible for the prevention of Aβ aggregation (Endres and Fahrenholz, 2012; Li et al., 2012). However, the Cd effects and mechanisms for amyloidogenic pathways are not well explored. Therefore, we next aimed to assess the main stress kinase p-JNK and their relationship with the amyloidogenic pathway, because recently we investigated that activated p-JNK leads to Aβ accumulation through activation of BACE-1 (Endres and Fahrenholz, 2012). Therefore, it was postulated that activated p-JNK might trigger BACE-1 in the Cd-exposed model. We found through immunoblotting that chronic Cd significantly activated p-JNK and BACE-1 levels in the brain compared to the control brain (Figure 6A). Confocal microscopy results also demonstrated that Cd significantly induced p-JNK immunofluorescence reactivity (Figure 6B). Further, we observed that Cd (10 μM) activated both p-JNK and BACE-1 in the HT22 cells, while the JNK-specific inhibitor (SP600125) rescued the activated p-JNK and BACE-1 immunoreactivity and co-localization in the Cd-exposed HT22 cells (Figure 6C), indicating that activated p-JNK has a role in BACE-1 activation.

### Cd Exposure Escalated the Amyloidogenic Pathway Through Activation of p-JNK

**Figure 7:**
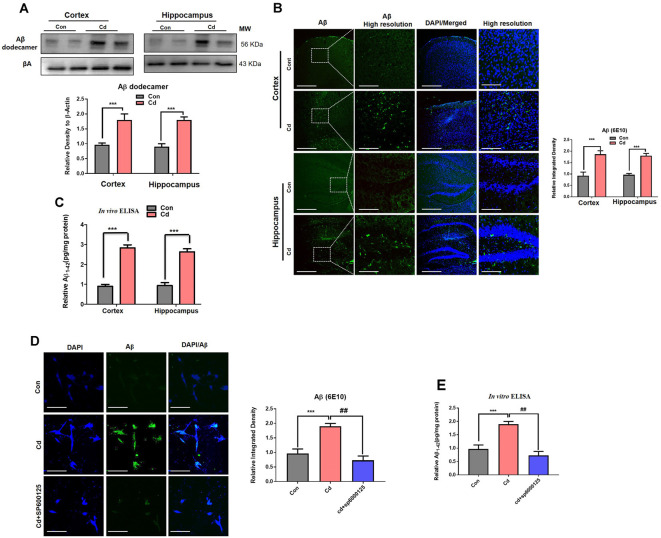
Cd exposure escalated the amyloidogenic pathway through activation of p-JNK. **(A)** Western blot images against Aβ oligomer in the cortical and hippocampal regions of the brain and their respective histograms. β-Actin was used as a loading control. The histograms represented as the means ± SEM (*n* = 7 mice/group) for three repeated and reproducible independent experiments. Significance = ***p < 0.001; Student’s unpaired *t*-test. See Supplementary Figure 4 for uncropped and original blots. **(B)** Representative immunofluorescence images of Aβ (green: FITC and blue: DAPI) in the cortical and hippocampal regions of the mouse brain. The histograms represented as the means ± SEM for *n* = 5 mice per group, and the number of independent experiments = 3. Magnification: 10×. Scale bar = 50 μm. Significance = ***p < 0.001; Student’s unpaired *t*-test. **(C)** The ELISA results for Aβ_1–42_ in the homogenates of the cortical and hippocampal regions of the brain. The histograms represented as the means ± SEM (*n* = 7 mice/group) for three repeated and reproducible independent experiments. Significance = ***p < 0.001; Student’s unpaired *t*-test. **(D)** The immunofluorescence images of Aβ (green: FITC and blue: DAPI) in the hippocampal neuronal HT22 cells exposed to Cd (10 μM) and SP600125 (20 μM). The data are indicated as the ±SEM for *n* = 5 images per group, and the number of independent experiments = 3. Significance = ***p < 0.001; ^##^*p* < 0.01; one way ANOVA followed by Tukey’s test. **(E)** The ELISA results for Aβ_1–42_ in the cell lysates of hippocampal neuronal HT22 cells exposed to Cd (10 μM) and SP600125 (20 μM). The data are indicated as the ± SEM for *n* = 5 per group, and the number of independent experiments = 3. Significance = ***p < 0.01; ^##^*p* < 0.01; one way ANOVA followed by Tukey’s test.

Activated BACE-1 acts through APP and converts it to Aβ, which accumulated in the aggregated form of the Aβ dodecamer. AβO and dodecamer are the most neurotoxic species of Aβ (Endres and Fahrenholz, 2012; Li et al., 2012; Ali et al., 2015, 2018; Beckhauser et al., 2016; Ng et al., 2016; Topic et al., 2016; Rehman et al., 2018; Shah et al., 2018). Therefore, we analyzed the APP and Aβ oligomer. Correspondingly, p-JNK and BACE-1 chronic Cd also escalated APP and the monomeric, oligomer, and dodecamer forms of Aβ in the brain compared to the control brain (Figure 7A). Aβ immunofluorescence indicated that Cd exposure increased the Aβ immunoreactivity in the brain mice compared to the control mice brain (Figure 7B). Further, to confirm immunoblotting/confocal microscopy results, we assessed the Aβ_1–42_ level through ELISA. The elevated level of Aβ_1–42_ was observed in the Cd-treated mouse brain compared to the control mouse brain (Figure 7C). To determine the underlying mechanism of JNK in the amyloidogenic processes, we used the JNK inhibitor and analyzed that SP600125 reduced the Aβ immunofluorescence reactivity in the Cd-exposed HT22 cells compared to Cd-exposed cells without SP600125 treatment (Figure 7D). Further, the *in vitro* Aβ_1–42_ ELISA results demonstrated that SP600125 significantly alleviated the Aβ_1–42_ level in the Cd-exposed HT22 cells compared to Cd-exposed cells without SP600125 treatment (Figure 7E). Overall, these results suggest that Cd induced activated ROS, suppressed antioxidant Nrf2, and activated p-JNK, which has a role in amyloid genic pathways. However, further mechanistic studies are needed to determine the exact underlying mechanism of these signaling in AD pathologies.

## Discussion

The major objective of this study was to explore the underlying mechanism of environmental factors such as cadmium chloride (Cd) in inducing AD-like pathological changes in the animal and cellular models. Cadmium exposure may induce an impaired antioxidant system, which may induce neurodegeneration and its associated memory impairment, correlating with human studies showing that people exposed to environmental factors would be more susceptible to aging. Both aging and environmental pollutants (Cd) are worldwide community problems since Cd is a major ubiquitously existing toxic food contaminant and is associated with lifestyle such as tobacco smoking habits and addictions (Kippler et al., 2012, 2016; Sanders et al., 2015; Richter et al., 2017; Ganguly et al., 2018; Gustin et al., 2018). Therefore, its underlying detrimental effects should be highlighted with a major focus on the old population, which are more vulnerable toward brain degenerations and its associated critical consequences such as cognitive impairments. We demonstrated that administration of chronic Cd to the mice induced memory dysfunctions, accumulation of oxidative stress and ROS, instigation of stress kinase p-JNK1, and suppression of antioxidant Nrf2/HO-1 pathways. Also, Cd exposure triggers activation of gliosis and other inflammatory receptors and mediators which induce neuroinflammation and consequently leads to neurodegeneration. These results represent proof of evidence that Cd acts as a potent environmental pollutant/contaminant, which accelerates aging-related AD pathologies, supporting previous epidemiological studies which have reported that Cd exposure and intoxication are remarkably correlated with AD-like pathological changes in humans (Min and Min, 2016; Peng et al., 2017).

Numerous earlier studies reported that environmental pollutants induced detrimental effects on human health and showed that Cd is mainly accumulated in the main organ of the body such as the liver, kidney, and heart (Horiguchi et al., 2013; Sanders et al., 2015; Kippler et al., 2016; Richter et al., 2017; Ganguly et al., 2018; Gustin et al., 2018). Nevertheless, recently several encouraging and compelling pieces of evidence reported that Cd crossed the BBB which accumulates in a different region of the brain. For Cd-induced neurotoxicity, the most vulnerable areas of the brain are the hippocampus and cortex, which are responsible for memory functions. Thus, accumulation of Cd mediates the impairment of spatial working memory and recognition memory, which our results also supported that chronic Cd exacerbates learning and memory deficits in the mice, which confirmed biochemical and immunohistochemical analyses through evaluation of pre- and postsynaptic markers. Our results were also supported by a few remarkable studies which showed that Cd is detrimental to dendritic spine density and synaptic plasticity as well as reduction of PSD95, which leads to memory defects (Antunes and Biala, 2012; Pulido et al., 2019). Chronic Cd exposure has been responsible for olfactory deficits along with cognitive impairments in young male mice. Further, a prospective cohort human study provided evidence that at the age of 10 years childhood Cd exposure might be responsible for impaired cognition in males. Similarly, Cd-exposed females exposed at the prenatal and childhood stages have been examined for behavioral impairment (Notarachille et al., 2014; Kippler et al., 2016; Gustin et al., 2018). These human cohort studies and other studies, as well as previous animal studies and our findings on preclinical animal models, provide evidence that Cd might cause gender-specific, genetically variable-based, and age-specific brain-associated diseases, but at the older age, it accelerates other aging-associated complications, which might be of more concern for the whole world, suggesting that as much as possible our old population should be less exposed to environmental pollutant/contaminant factors to reduce aging-associated brain disorder burden, particularly dementia, a major health problem to the community.

Recently, Huat et al. critically reviewed that overwhelming evidence showed that metals, e.g., lead, methyl mercury, and Cd, are the potent inducers of oxidative stress and elevated ROS accumulation, which leads to cellular aberrant homeostasis (Kim et al., 2018; Huat et al., 2019). Potent pro-oxidant and ROS stimulant Cd also suppressed the endogenous antioxidant system. Cd has been involved in the suppression of Nrf2/HO-1 pathways (Kensler et al., 2007; Agnihotri et al., 2015; Khan et al., 2019; Branca et al., 2020), supported by our studies that subacute administration of Cd reduced the Nrf2/HO-1 level in the aged mouse brain. Nevertheless, further studies are needed to explore the modulation pattern of Nrf2/HO-1 in response to acute and subacute administration of Cd or other environmental pollutants at different periods of experimental models. Of note, Nrf2 stimulation has a role in the suppression of BACE-1, so it is possible that the reduced level of Nrf2 also favors the p-JNK activation of BACE-1 and consequently the production and accumulation of Aβ (Rehman et al., 2018; Bahn and Jo, 2019; Bahn et al., 2019).

Epidemiological studies reported that Cd levels in the blood plasma are implicated in the pathologies of AD and its associated mortality in people, supported by several studies that Cd has a key role in the accumulation of Aβ in the brain (Ashok et al., 2015; Min and Min, 2016; Peng et al., 2017; Kim et al., 2018; Huat et al., 2019). Notably, the combination of Cd with other environmental elements (arsenic and lead) robustly increased APP, BACE-1, and PSEN1 which led to Aβ aggregation, supposing a synergistic effect of Cd with other environmental factors which trigger AD pathologies (Ashok et al., 2015; Zhou et al., 2015). During elevated stress kinases, the most important stress kinase is activated p-JNK, which has been implicated in the production of amyloidogenic pathways through activation of β-secretases, which leads to cleavage of the β-APP site, resulting in triggered misfolded aggregation of the Aβ oligomer and accumulation of Aβ plaques, and inhibition of p-JNK reduced this amyloidogenic pathway (Ashok et al., 2015; Zhou et al., 2015; Rehman et al., 2018). The previous studies demonstrated that chronic Cd accelerates the basic hallmarks of AD, which further mediates AD-associated pathologies. Of note, our results demonstrated that subacute and chronic Cd administration increased the Aβ dodecamers in the aged mouse brain as compared to the nontreated mice. It has been worth describing that Aβ dodecamers are associated with aging and have been found at higher levels in aging patients with mild cognitive dysfunctions (Lesn et al., 2013). These studies were further supported by a study that administration of Aβ dodecamer to healthy rats induced memory deficits (Lesné et al., 2006), indicating that they mediate age-associated AD pathologies.

The effect of environmental contaminants on ROS/oxidative stress-mediated AD pathologies and their relationship with metabolic stress should be investigated. This is because the lifestyle and brain metabolism-associated AMP-activated protein kinase (AMPK) and silent information regulator 1 (Sirt-1) signaling have a key role in maintaining cellular homeostasis and protecting the cells against external and internal stress or insults and have a protective role in AD pathologies (Shah et al., 2017; Gu et al., 2018; Yoon et al., 2018; Ali et al., 2021). AMPK act as a fuel sensor and master regulator of cellular energy and plays a vital role in stress conditions and regulates the global cellular energy homeostasis in the brain as well as in the peripheral system. Likewise, Sirt1 has a promising role in cell longevity, senescence, and cellular signaling, and homeostasis which prevents aggregation of misfolded proteins, neuroinflammation, and neurodegeneration (Zhang et al., 2011; Hardie et al., 2012; Shah et al., 2017). Further, aging is the major risk factor for AD and its associated dementia. With the progression of aging, immunosenescence takes place in the CNS which increases vulnerability to diseases (Cribbs et al., 2012; Maqbool et al., 2013). The overstimulated proinflammatory has been produced in the aged brain. Therefore, it is important to highlight the effect of subacute administration of Cd on these signaling pathways in the different time periods of treatment associated with aging.

## Conclusions

**Figure 8:**
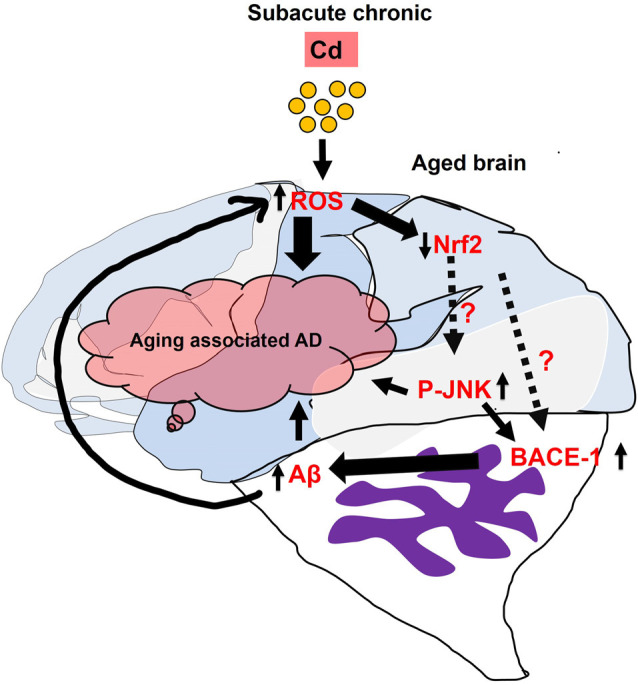
Represents the generalized overview and possible mechanistic approach of subacute Cd administration which induced Alzheimer’s disease (AD)-associated pathologies in the aged mice’s brain. 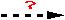 Represented that direct correlation and mechanism between the Nrf2 and p-JNK as well as theirrelationship with BACE-1 should be determined comprehensively in *in vitro* and *in vivo* models.

In conclusion, we demonstrated that Cd is a potent environment neurotoxic factor which induces AD-related pathological features in the mice’s brain. We investigated that administration of subacute and chronic Cd to the mice induced elevated ROS/oxidative stress, suppressed the antioxidant Nrf2/HO-1 markers, and evoked p-JNK1 which might trigger the activation of the Aβ pathologies and learning and memory impairment (Figure 8). Of note, the direct correlation and mechanism between Nrf2 and p-JNK as well as their relationship with BACE-1 should be determined comprehensively in *in vitro* and *in vivo* models. Therefore, Cd exposure shows a critical health problem that threatens the quality of life and must be considered a serious pathophysiological risk factor for neurodegenerative diseases. We believe that this preclinical animal finding unveils cellular mechanisms for targeting Cd-exacerbated neurodegeneration, but also representing a paradigm for people exposed to Cd and suggesting to avoid environmental factors in their late life to mitigate Alzheimer’s pathology-associated dementia. Most importantly, one of the interesting studies described the co-relation and synergetic effect of the gene–environment (ApoE4 and Lead) interaction which impaired cognitive behavior in animals (Engstrom et al., 2017). Further, in the future, we warrant and suggest that the gender and genetic variability bases particularly for late-onset AD associated with ApoE4 carrier-based studies as well as in the context of lifestyle should be designed and investigated in the aging preclinical animal models of neurodegenerative diseases. These models and translation research will directly impact our community to study epidemiological and retrospective approaches in the population and would be useful for the researchers to further elucidate the underlying mechanism of subacute administration of Cd, which will lead to identify the cellular therapeutic targets or the prevention and management of environmental contaminant-associated brain degeneration.

## Data Availability Statement

The original contributions presented in the study are included in the article/Supplementary Material, further inquiries can be directed to the corresponding author.

## Ethics Statement

The animal study was reviewed and approved by The animal’s ethics committee of the Division of Applied Life Sciences, Gyeongsang National University, South Korea (Approval ID: 125).

## Author Contributions

TA designed the research, wrote the manuscript, and rearranged all the data. AK, MI, and SA performed the immunoblotting. SIA and TA performed immunofluorescence and ELISA. MI, TA, and AK performed the behavioral study, calculation, and data analysis. TA, JP, and HL conducted all *in vitro* work and analyzed the data. AK provided the concept of project and contributed in reviewing and editing the whole manuscript. MK reviewed and approved the manuscript, and all authors reviewed the manuscript. MK is the corresponding author and holds all the responsibilities related to this manuscript. All authors contributed to the article and approved the submitted version.

## Conflict of Interest

The authors declare that the research was conducted in the absence of any commercial or financial relationships that could be construed as a potential conflict of interest.
