# Supplementary material for: Cadmium, an Environmental Contaminant, Exacerbates Alzheimer’s Pathology in the Aged Mice’s Brain
Source: Front Aging Neurosci. 2021 Jun 24;13:650930. doi: 10.3389/fnagi.2021.650930 (PMC8263901; doi:10.3389/fnagi.2021.650930)
Supplement: Supplementary file 1 [file Table_1.DOCX]

Supplementary Material

**Cadmium, an Environmental Contaminant exacerbates Alzheimer’s Pathology in the Aged Mice’s Brain**

**Tahir Ali^1,2,3+^, Amjad Khan^1 +^, Sayed Ibrar Alam^1+^, Sareer Ahmad^1^, Muhammad Ikram^1^, Jun**

**Sung Park^1^, Hyeon Jin Lee^1^, Myeong Ok Kim^1^***

^1^ Division of Applied Life Science (BK 21), College of Natural Science, Gyeongsang National University, Jinju, 660-701, Republic of Korea.

^2^ Calgary Prion Research Unit, Department of Comparative Biology & Experimental Medicine, Faculty of Veterinary Medicine, University of Calgary, Calgary, Alberta, Canada.

^3^Hotchkiss Brain Institute, Cumming School of Medicine, University of Calgary, Calgary, Alberta, Canada.

*Correspondence: mokim@gnu.ac.kr (M.O.K); Tel: (+82-55-772-1345)

**+**These authors equally contributed to this paper.

**Keywords: Aging; Alzheimer’s disease; Cadmium; Reactive oxygen species; antioxidant genes Nrf-2/HO-1.**


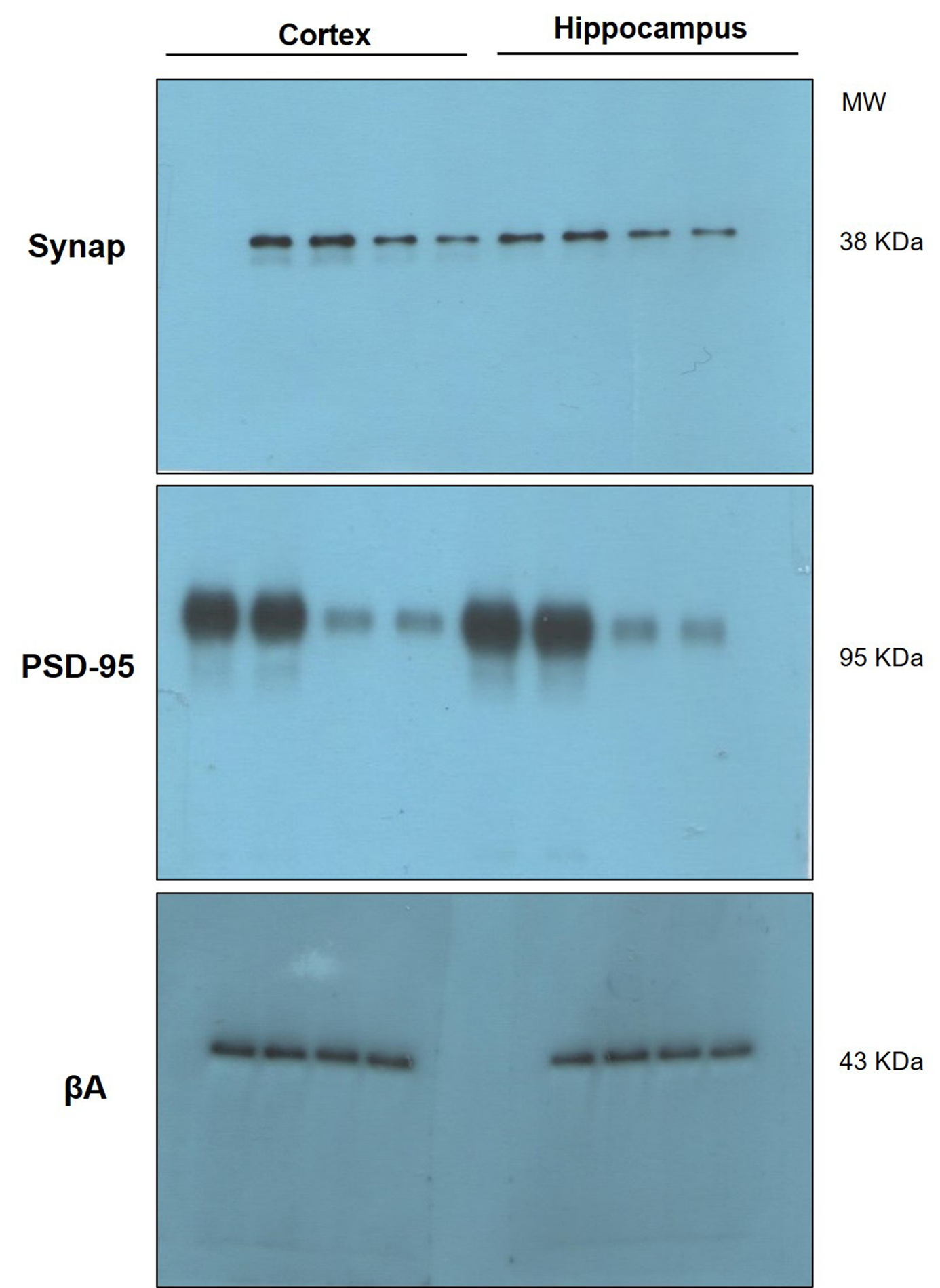


Figure S1. Uncropped scans of western blots included in Figure 3a.


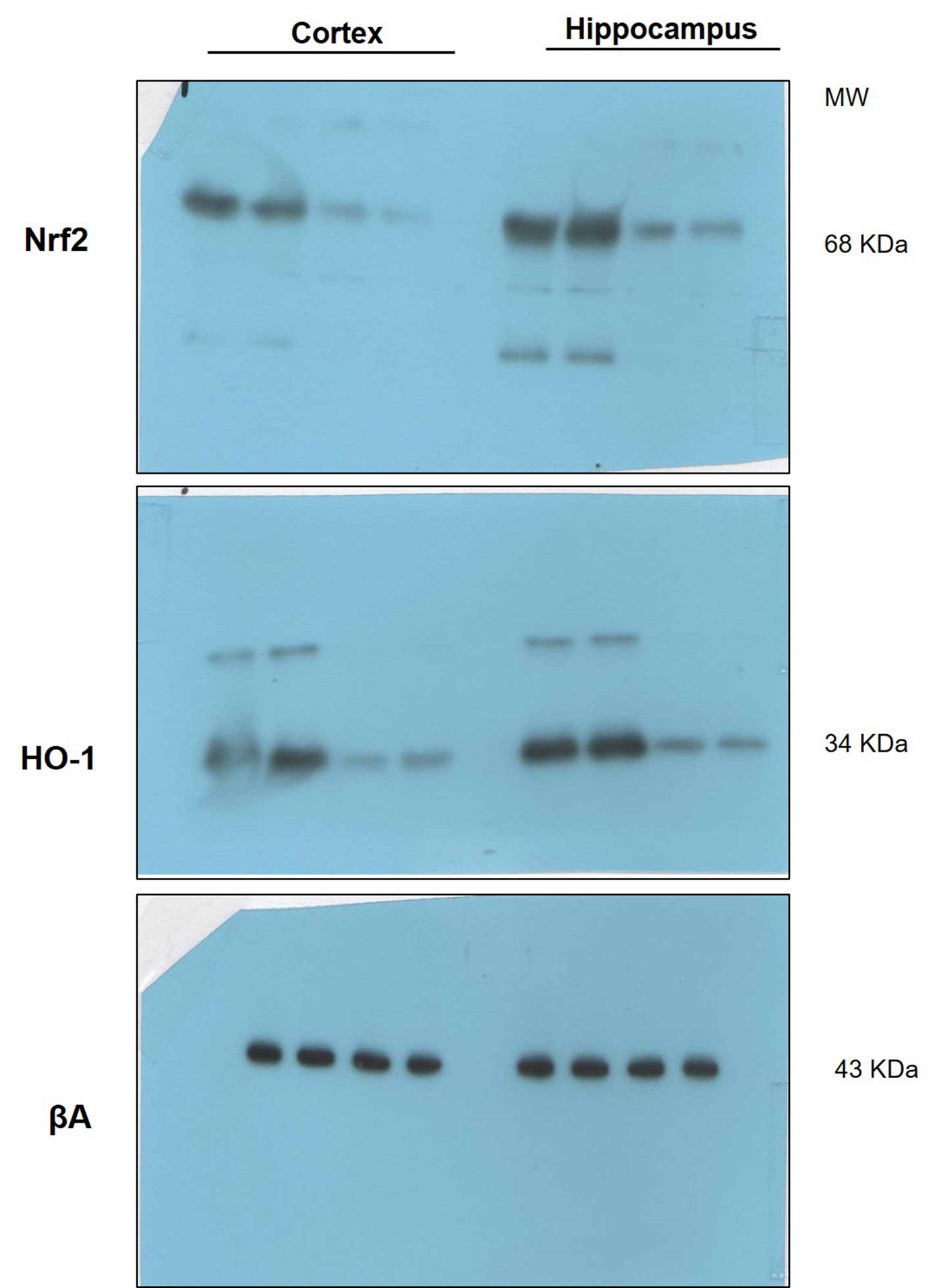


Figure S2. Uncropped scans of western blots included in Figure 5a.


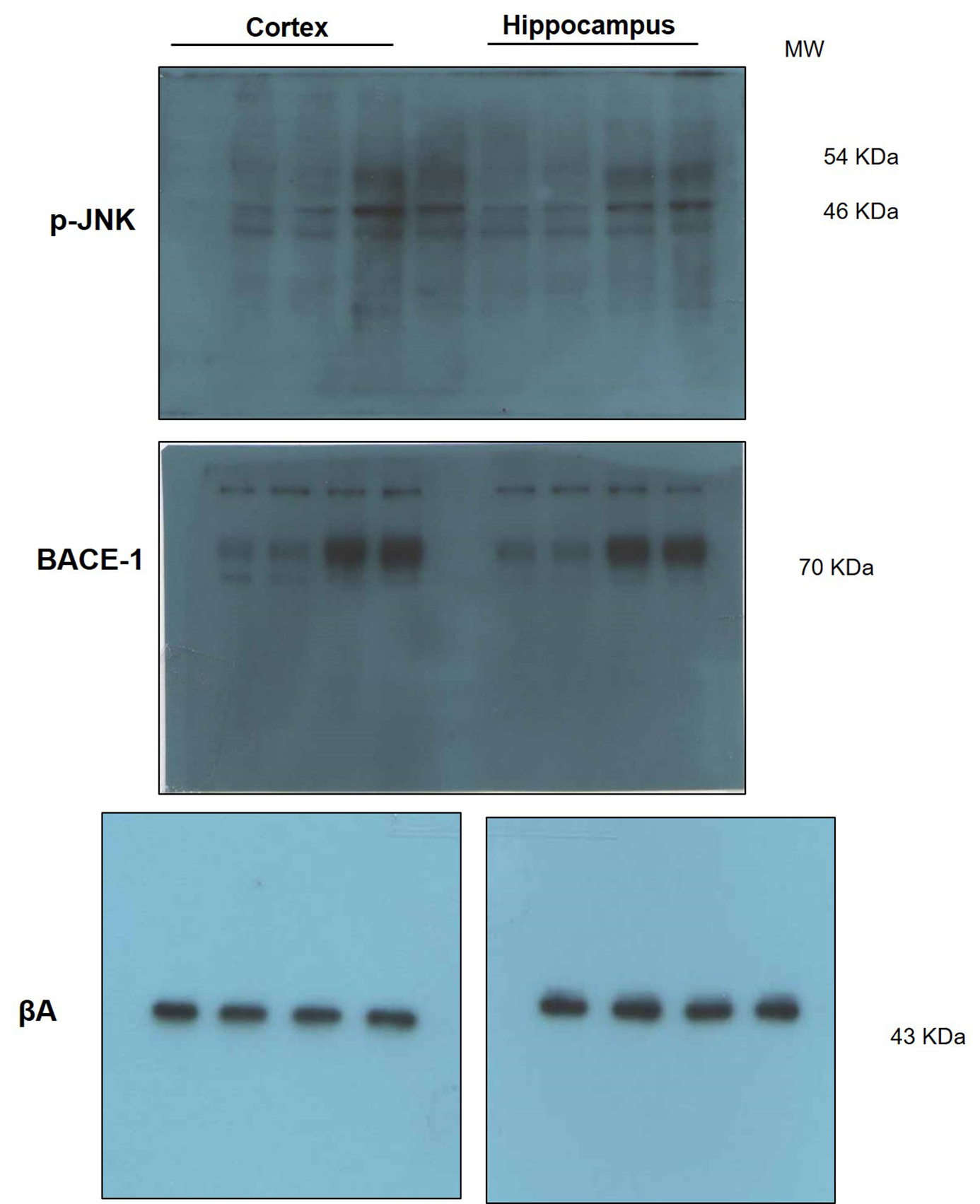


Figure S3. Uncropped scans of western blots included in Figure 6a.


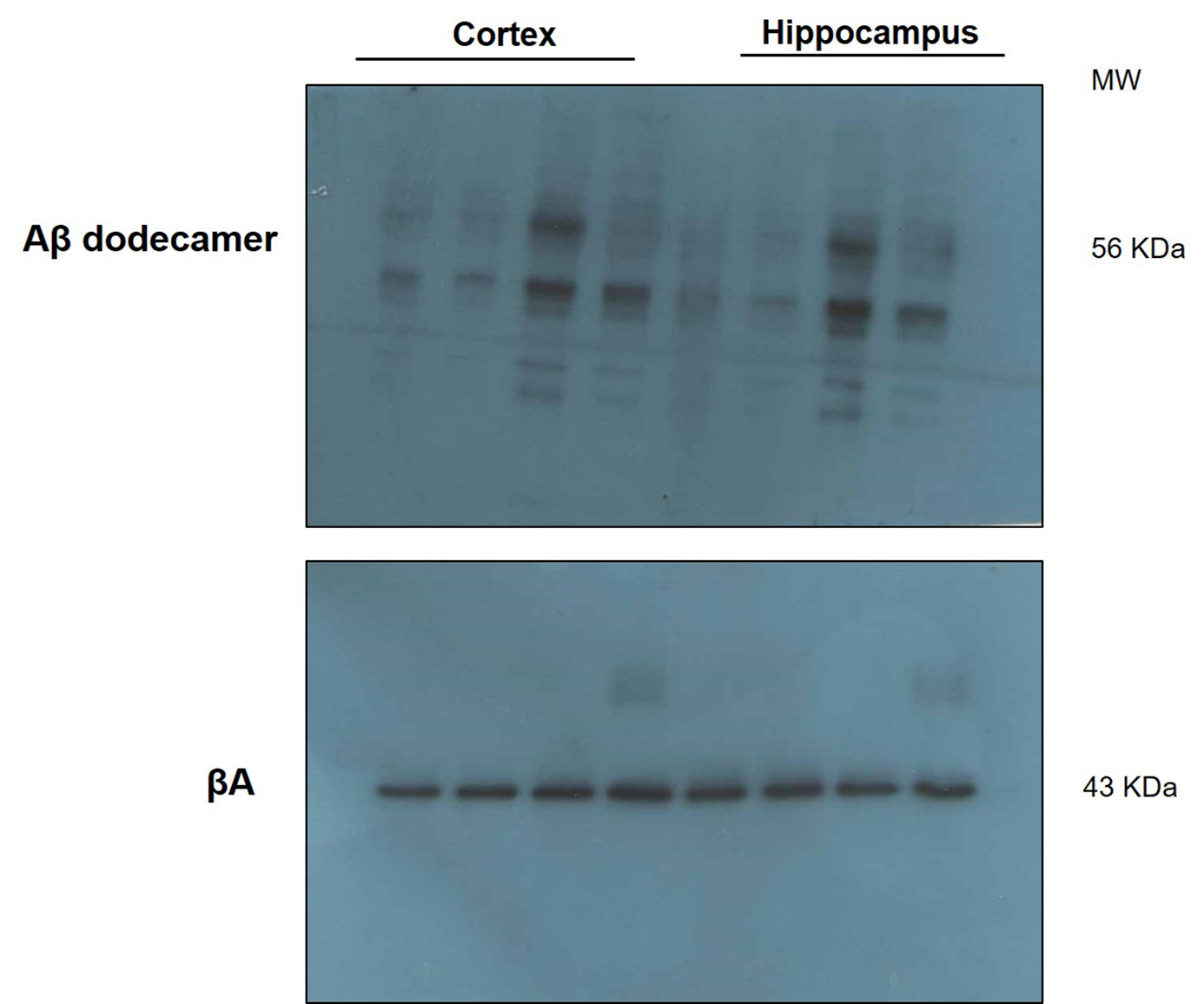


Figure S4. Uncropped scans of western blots included in Figure 7a.
